# Olive Yield and Physicochemical Properties of Olives and Oil in Response to Nutrient Application under Rainfed Conditions

**DOI:** 10.3390/molecules28020831

**Published:** 2023-01-13

**Authors:** Ermelinda Silva, Alexandre Gonçalves, Sandra Martins, Cátia Brito, Helena Ferreira, Luís M. M. Ferreira, José Moutinho-Pereira, Manuel Ângelo Rodrigues, Carlos M. Correia

**Affiliations:** 1 Centre for the Research and Technology of Agro-Environmental and Biological Sciences (CITAB), University of Trás-os-Montes e Alto Douro, 5000-801 Vila Real, Portugal; 2 Association BLC3—Technology and Innovation Campus, Centre Bio R&D Unit|North Delegation, Rua Comendador Emílio Augusto Pires, 14, Edifício SIDE UP, 5340-257 Macedo de Cavaleiros, Portugal; 3 Collaborative Laboratory Mountains of Research (MORE), Brigantia Ecopark, 5300-358 Bragança, Portugal; 4 Inov4Agro—Institute for Innovation, Capacity Building and Sustainability of Agri-Food Production, University of Trás-os-Montes e Alto Douro, 5000-801 Vila Real, Portugal; 5 Department of Animal Science, University of Trás-os-Montes e Alto Douro, Quinta de Prados, 5000-801 Vila Real, Portugal; 6 Mountain Research Center (CIMO), Polytechnic Institute of Bragança Campus de Santa Apolónia, 5300-253 Bragança, Portugal; 7 Associated Laboratory for Sustainability and Technology in Inland Regions (LA SusTEC), Polytechnic Institute of Bragança, Campus de Santa Apolónia, 5300-253 Bragança, Portugal

**Keywords:** *Olea europaea* L., nitrogen, phosphorus, potassium, boron, fruit and oil quality, phenolic quantification, oil storage, sensorial analysis

## Abstract

The effects of mineral fertilizers on the physicochemical properties of olives and oil under rainfed conditions is scarce. In this three-year study, the results of a nitrogen (N), phosphorus (P), potassium (K) and boron (B) fertilization trial carried out in a young rainfed olive grove and arranged as a nutrient omission trial are reported. The control consisted of the application of N, P, K and B (NPKB) and four other treatments corresponded to the removal of one of them (N0, P0, K0 and B0). Olive yield and several variables associated with the physicochemical properties of olives and oil were evaluated. The NPKB treatment increased olive yield compared to the treatment that did not receive N (N0). Although dependent on the climate conditions of the crop season, the NPKB treatment increased fruit weight and the pulp/pit ratio and its fruits tended to accumulate more oil than K0. However, the phenolics concentrations on fruits and oil tended to be lower. All olive oil samples were classified in the “extra virgin” category and all showed a decrease in its stability between 3 and 15 months of storage, regardless of treatment, especially in N0, P0 and B0 treatments. The results of the sensorial analysis indicate that all the oils fell into the medium fruitiness and greenly-fruity category. Only in P0 and B0 were defects detected, namely muddy sediment. Thus, this study seems to indicate the importance of N application, but also a balanced nutrient application and that further studies are needed, given the difficulty in finding clear trends in the response of measured variables to fertilizer treatments.

## 1. Introduction

Olive (*Olea europaea* L.) is the perennial tree species occupying the largest hectarage in the world [1], being particularly dominant in the cultivated landscape of vast areas of the Mediterranean basin. Portugal is the ninth largest producer in the world, with two dominant systems: high-density irrigated hedgerow orchards in the South and traditional rainfed orchards in the Northeast of the country, where the high number of varieties, associated with the high economic, social, and environmental value of the crop in a mountainous and disadvantaged region, constitutes important genetic and landscape heritage that must be preserved.

Olives are rich in polyphenolic compounds that provide numerous health benefits [2], mainly oleuropein, demethyloleuropein, hydroxityrosol and verbascoside. Olives are also rich in monounsaturated fatty acids (MUFA), mostly oleic acid (47–84%) and palmitoleic acid (0.3–3.5%), and polyunsaturated fatty acids (PUFA) such as linoleic (5–10%) and linolenic (0.2–1.5%) acids. Likewise, other important compounds are present in low amounts such as sugars, carotenoids, tocopherols, chlorophylls and anthocyanins [3,4]. On the other hand, olive oil is an important source of heart-healthy fats along with vitamins. It is also associated with several beneficial effects on human health due to its balanced fatty acid composition and antioxidant, antimicrobial, anti-inflammatory, antidiabetic, anticarcinogenic and cardioprotective properties [5,6], and contributes to a more balanced diet, being a fundamental element for those who want to maintain a healthy lifestyle [7]. It is mostly composed by triacylglycerols (98%), free fatty acids, pigments, phenols, tocopherols, sterols, phospholipids, waxes, squalene, and other hydrocarbons [8]. The phenolic compounds present in olive oil have an important role in sensorial attributes, particularly spicy, astringent and pungent sensations, as well as in the oil quality and stability [9]. The olive oil properties may change with several agronomic (irrigation, fertilization, date of harvest and fruit maturation, cultivars), technological (postharvest storage and extraction process) and edaphoclimatic factors [8,10].

Although an adequate application of fertilizes can positively affect photosynthesis and whole-tree physiology, growth and olive yield [9,11,12,13,14], its effect on oil quality is scarce and inconsistent, especially under rainfed conditions [15,16]. Still, some trends can be highlighted. Regarding N, most studies indicate that N fertilization reduced the concentrations of phenolics in the oil, either under rainfed or irrigation management [15,16,17], indicating, according to the protein competition model, that phenylalanine preferentially flows into protein synthesis rather than toward synthesis of phenylpropanoids [15]. On the other hand, free fatty acids (FFAs) increased with N application, along with and a positive correlation with N in fruits [15,17].

Regarding P application, a minor influence on quality has been reported [16], although a decrease in polyphenols in response to high P fertilization was observed [18]. However, the effect of P on oil quality was mainly indirect, since high P availability increased N accumulation [15,16]. In addition, Erel et al. [15] found no significant effects of P levels on FFAs. Meanwhile, the role of K is more controversial [16]. K nutritional level had only a minor effect or no effect at all on oil quality traits such as polyphenols or fatty acids contents, probably because unlike N and P, K is not an intrinsic mineral in oil or in any organic tissue [15,18]. Furthermore, fertilization with micronutrients has been even less studied. An optimum B application has been shown to improve oil yield [19] and quality, either by improving fatty acid composition, total phenols content and major volatile compounds [20], or by decreasing the acid and peroxide values [19]. Nonetheless, a decrease in total phenols and o-diphenols has been observed after foliar fertilization with boron (B), magnesium (Mg), manganese (Mn) and sulfur (S), due to the B action [21]. Thus, it seems clear that more field trials are needed to evaluate the effects of fertilizers application on oil and olive quality, especially in rainfed olive groves. The aim of this study was to evaluate the effect of the application of N, P, K and boron (B) on olive yield, as well as on the olives and oil quality, based on a nutrient omission trial, from which the combined effect of all nutrients or each one separately can be compared. N, P, K and B were the nutrients used in this study, since they are the four nutrients that olive growers of northeast of Portugal apply most regularly to olive trees.

## 2. Results and Discussion

### 2.1. Crop Yield, Tree Nutritional and Water Status, Photosynthesis and Fruit Physicochemical Characterization

**Figure 1:**
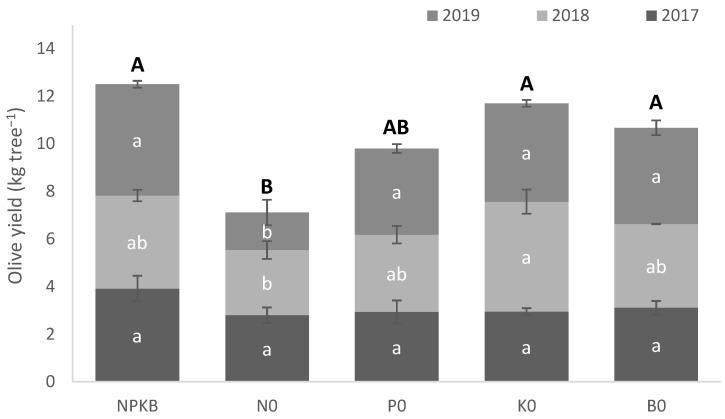
Olive yield from trees supplied with NPKB (control) and without N (N0), P (P0), K (K0) and B (B0) in 2017, 2018 and 2019. Vertical bars represent the standard errors. Within each year (lower case letters) and accumulated total (upper case letters), means followed by the same letter are not significantly different by Tukey’s HSD test (α = 0.05).

The NPKB control increased (*p* < 0.05) the accumulated olive yield (2017–2019) compared to the N0 treatment (Figure 1). Nevertheless, it should be pointed out that differences between treatments were only recorded in 2019. The K0 and B0 treatments also showed higher (*p* < 0.05) accumulated olive yields than the N0 treatment. P0 showed average accumulated olive yield above N0, but not significantly different. However, in this case, differences (*p* < 0.05) between P0 and N0 treatments were also found in the last harvest (2019).

At the end of the growing season, leaf N concentration was significantly lower in the N0 treatment in comparison to N fertilized treatments (Table 1). Leaf K levels were also the lowest in the K0 treatment, significantly different to N0 treatment. Leaf B levels were significantly lower in the B0 treatment when compared to the leaf B levels in the treatments receiving B as a fertilizer. No significant differences between treatments were found for leaf levels of P, Ca, Mg, Fe, Zn, Cu and Mn.

Previous studies have shown that fertilizer application can play an important role in olive yield, but not all studies have found consistent results [13,14,23,24]. For instance, Erel et al. [25] reported that fruit set and olive yield was negatively affected by N deficiency but not by K. Under N deficiency, these authors observed a reduction in olive yield, probably due to a decrease in protein biosynthesis, which is important for the development of the inflorescences. A reduction in the number of flowers and fruit set was commonly observed in poorly N-fertilized olive orchards [25,26]. Rodrigues et al. [26] reported a yield reduction in trees maintained for 4 years without N in comparison to those regularly fertilized. Haberman et al. [27] suggested that trees under N deficiency are also more susceptible to alternate bearing. Ferreira et al. [28] did not find an olive yield response to P applied to soil in pot experiments and in a field trial. The authors suggested that local farmers could reduce P application without significant risk of lost productivity. The olive tree also tends to not respond to K fertilization. Ferreira et al. [11] did not observe a decrease in leaf K levels in the non-K-fertilized control and this was likely the reason why they did not find significant differences between treatments in olive yield. Erel et al. [15] were able to find a decrease in olive yield in a container experiment, but only after a prolonged and severe K deficiency. Regarding B, previous studies have shown that nutrient application tends to increase leaf B levels and also crop productivity [12,26,29]. In this study, leaf N, K and B levels clearly decreased in N0, K0 and B0 treatments, but the values were close to the lower limit of the sufficiency range. The nutritional stress to which the plants were exposed was not very severe and only in the case of N did it negatively influence productivity.

**Figure 2:**
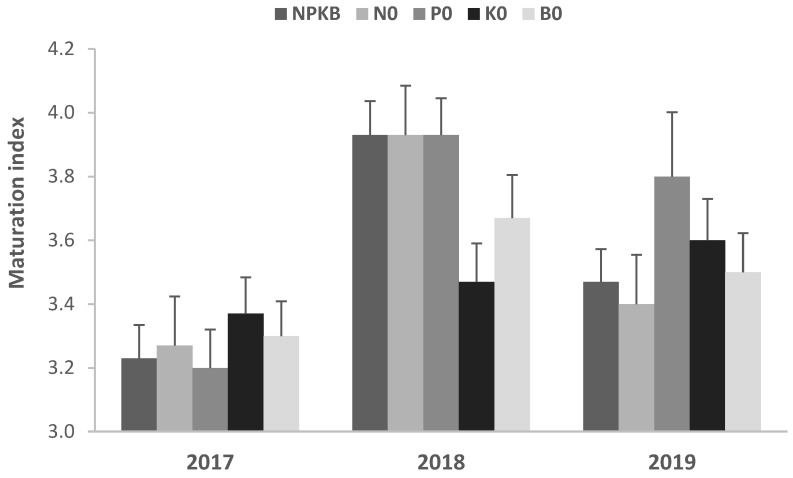
Maturation index of olives from trees supplied with NPKB (control) and without N (N0), P (P0), K (K0) and B (B0) in the harvests of 2017, 2018 and 2019. Vertical bars represent the standard errors.

The ripeness stage of the olives, evaluated as MI, varied, but not consistently with the year (Figure 2). In trees with high fruit load, maturation tends to be delayed compared to trees with low production [30]. However, in this study the production of olives in the three years was not very dissimilar in most treatments, meaning that a clear alternate-bearing pattern was not observed, and the decision on the harvest date also did not consider the state of maturation of the fruits, so it is not possible to establish a relationship between fruit load and MI. Moreover, fertilizer application itself does not seem to affect fruit maturation [31], which is consistent with the results obtained in our study. The main reasons that generally justified the higher MI in 2018 were the later harvest and the higher average temperature/heat accumulation (GDD) in September. As reported by Mafrica et al. [32], lower temperatures slow down fruit growth and development and delay ripening.

**Figure 3:**
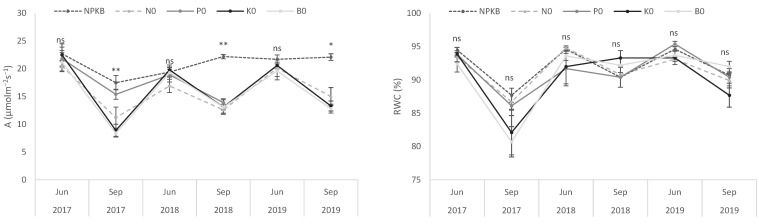
Net photosynthetic rate (A) and relative water content (RWC) of leaves from olive trees supplied with NPKB (control) and without N (N0), P (P0), K (K0) and B (B0) in two periods of 2017, 2018 and 2019. Vertical bars represent the standard errors.

Regarding the biometric variables, significant differences between treatments were only found in 2018 (Table 2). NPKB plants showed heavier (*p* < 0.05) fresh fruits than those of the other treatments. This was due to the greater pulp FW in NPKB plants, since pit FW did not differ between treatments, which also resulted in a significant higher pulp/pit ratio. The results are consistent with those obtained in previous reports [31], where NPK fertilizers were found to increase the average fruit weight and pulp/pit ratio. The decrease in fruit FW, pulp FW and pulp/pit ratio observed in the treatment that did not receive N and in the treatments K0 and B0 was probably due to a reduction in photosynthetic capacity, which may reduce the availability of photosynthates [33]. In fact, net photosynthesis data presented in Figure 3 showed lower values in P0 and, especially, in N0, K0 and B0 treatments in September, usually a period of important fruit growth under rainfed conditions. These responses were not associated with leaf water status as the application of fertilizers did not affect RWC values, meaning that the effects on photosynthesis were more associated with leaf nutrient levels and their role in the photosynthetic process.

### 2.2. Nutritional Value of Olives and Oil

Most of the nutritional parameters of olives were influenced by fertilizer treatments, the exception being OM (Table 3). Unfortunately, few studies have evaluated the effects of fertilizer application on olive nutritional variables. Conte et al. [34] reported that nutritional properties of olives can be influenced by agronomic practices such as irrigation and fertilization and also by the fruit ripening stage. Fruit ripeness, for instance, may affect several nutritional variables of olive fruits such as oil content, total soluble solids, total polyphenol and antioxidant activity [34]. In the present study, the olives from treatment B0 had lower dry matter values compared to the other treatments, which is consistent with the results reported by Delıboran et al. [35], who found a decrease in olive dry matter in treatments in which B was not applied.

The main components of olives are fat, fiber, proteins and sugars [36,37,38]. In our study, dietary fiber content varied between 11 and 14% (Table 3), which agrees with the values obtained by Jiménez et al. [37] (about 10–15%). Olives in the P0 treatment showed lower (*p* < 0.05) dietary fiber in both years. The dietary fiber content of olives is composed of pectin, cellulose and lignin that can vary throughout the ripening stage [39]. As fiber intake has a protective effect against heart disease [36], consumption of olives from plants that are less fertilized with P appears to confer less health protection.

The protein content varied with the fertilizer treatments between 0.9 and 4% (Table 2). Lanza [38] also observed low protein content in olives; however, the nutritional quality of olives remained high due to the presence of essential amino acids such as threonine, valine, leucine, isoleucine, phenylalanine, lysine, arginine, histidine and tyrosine. Although in this study the consistency in the results of the protein content was low, when the effects of the treatments in the two years were compared, the higher values were always observed in B0 treatment.

Oil content also varied greatly between treatments in the two years of study (Table 2). K0 showed a clear tendency for lower oil content. This result may be related to the higher demand for K from the fruits, mainly during oil accumulation [40]. Regarding soluble sugars, a tendency for higher contents was observed in the olives of the K0 treatment (Table 3). Considering these results and that the precursor of biosynthesis of fatty acids is acetyl-CoA, derived from a catabolism of sugars such as glucose and fructose [41], in general, our data corroborate the sugar-oil negative linear relationship presented by Migliorini et al. [42], also confirming that sugar concentration may be considered as a direct index for the peculiar biochemical process of oil accumulation during olive ripening. Meanwhile, the low oil content obtained in 2018 was mainly related to high rainfall, especially in October and November, which contributes to very low dry matter content. High fruit moisture makes it difficult to extract oil from the olive, interfering with oil coalescence during the malaxation process [43].

### 2.3. Phenolic Composition of Olives and Oil

The contents of TPC, o-DP, Fl and TCA in olives are shown in Table 4. Despite the reduced consistency in the results of the two years, the olives from the NPKB treatment had generally low TPC, o-DP and Fl and low TAC. On the contrary, an increase in phenolic compounds, mainly o-DP and Fl, was observed in P0 treatment in both years. In the K0 treatment, an increase in the Fl content was also observed. The composition of the fresh pulp of olives contains 1–3% of phenolic acids, including phenolic alcohols, flavonoids and secoiridoids. The phenolic composition is very complex and depends on the fruit maturation stage, cultivar and growing season [44]. During olive ripening there is an increase in the content of phenols to a maximum level in the mid-pigmentation phase, followed by a sharp decrease as ripening progresses [45]. El Riachy et al. [46] observed that a decrease in phenolic compounds was associated with the olive ripening process. However, a decrease in phenolic compounds in both years of study was verified only in the NPKB treatment that has MI similar to other treatments such as P0 and N0. The higher levels of total phenols, flavonoids and TAC recorded in fruits in 2017 relatively to 2018 may be justified by the earlier harvest and the slightly lower MI and, especially, by the distinctive environmental conditions that include more severe summer stress in 2017 that exacerbates the oxidative stress, promoting the biosynthesis of this group of antioxidant compounds. As can be seen in Table 5 the accumulated precipitation was much lower in 2017 than in 2018, including in October and November, close to the harvest time. Moreover, 2017 presented a higher mean temperature and superior number of hot days, particularly in June and July. These data justify the higher accumulation of phenolic compounds in 2017, in agreement with other studies, which reported that stressful conditions increase the accumulation of phenolic compounds in olive fruits [47]. Eventually, the differences could have been even more evident if there had not been a high number of frost days (4) and lower minimum temperatures immediately before the 2017 harvest. Temperatures below 0 °C result in the freezing of extracellular water, and the thermodynamic equilibrium is achieved either by cellular dehydration and continued extracellular ice formation, or by intracellular ice formation. These effects seriously damage cell membranes, leading to cell death and the oxidative degradation of cell contents such as phenolic compounds, due to the contact between enzymes and their respective substrates [48].

Regarding the oil phenolic composition, a similar trend was observed for the olives in the NPKB treatment (Table 4). The oil from the NPKB treatment showed low TPC, o-DP and Fl contents and TAC in both years. Baiano et al. [50] reported a strong positive correlation between phenolic compounds and antioxidant activity. Phenolic compounds contribute to color, flavor and shelf life and also to the stability of extra virgin olive oils due to radical scavenging [51]. Thus, the higher level of phenols observed in the oil of the less fertilized treatments may have conferred greater protection against oxidation. In contrast to that observed in olives, higher values of total phenols, flavonoids and TAC in olive oil, as well of ortho-diphenols, were recorded in 2018, meaning that a high percentage of phenolics compounds was transferred from olive pulp to oil in 2018. To explain the different trend in the transference of phenolic compounds to oil between years, different hypotheses can be raised, including changes in enzymes activities during the pressing and malaxation steps, and/or changes in the transference of specific phenolics presented in fruits and olive stones and lignans after whole olive fruits crushing and malaxation, contradicting the deduction that more phenols in fruits would lead automatically to more phenols in oil [52].

### 2.4. Olive Oil Quality

Fertilizer application can have a marked effect not only on olive tree performance, but also on olive oil quality [15]. Oil quality is usually estimated by the degree of primary oxidation (peroxide index and K232) and secondary oxidation (K270 and ∆K) [50]. These quality parameters established by the European Union (EU) are extremely important to ensure the good quality of olive oil [53].

All olive oil samples analyzed in this study are classified in the “extra virgin” category, presenting low mean values of free acidity (<0.8%), peroxide index (<20 meq O_2_ kg^−1^), K270 (<0.22) and K232 (<2.5) [54]. However, some statistical differences were observed in the peroxide index, K270 and ∆K (Table 6). Oil from NPKB treatment showed a higher (*p* < 0.05) peroxide index compared to N0, K0 and B0, but only in 2017. Despite the increment of peroxide index observed in NPKB, this value was below the threshold value (<20 meq O_2_ kg^−1^) established in Regulation 1348/2013 [54]. Olive oil from K0 treatment showed high K270 and ∆K. These variables measure the secondary oxidation products and are used as a criterion to establish the degree of degradation of the oil [55]. Meanwhile, the higher values of free acidity, peroxide index and oil UV extinction coefficients noticed in 2018 relatively to 2017 were related with the slightly higher maturation index and with high values of temperature (September) and rainfall during the last fruit growth months (October and November). In other studies, it was also observed that at advanced stages of olive maturity, the percentage of free acidity increases due to the increase in enzymatic activity, specifically by lipolytic enzymes, favored by olive tissue damages [56], while increments of K232 and K270 were reported under high autumnal temperature and rainy periods [57], respectively.

### 2.5. Olive Oil Storage and Sensorial Analysis

The quality of the olive oils produced in 2018 was evaluated after 15 months of storage (Table 7). A decrease in oil stability was observed regardless of the treatment, with a reduction in PV and an increase in K232 and K270. Between treatments, P0 and K0 presented higher K270 in relation to the NPKB treatment. In general, after 15 months of storage, there was an increase in K232 and K270 in all oils, reaching values above the limit established in EU Regulation 1348/2013 [54], which indicates a clear oil degradation. It is well-known that storage time and conditions can induce changes in olive oil quality [58]. In situations of exposure to oxygen and light, high temperatures and the presence of trace metal ions can accelerate lipid oxidation and then shorten the shelf life of the product [59]. More specifically, the stability of the oils depends on the oleic/linoleic acid ratio and on phenolic profile [21]. During storage, changes in the profile of phenolic compounds due to the hydrolytic processes of oleuropein and oxidation of o-DP cause degradation of oil quality and affect the sensory analysis [8]. It is important to mention that the oils from the N0, P0 and K0 treatments were more exposed to degradation during storage time than the oil from the NPKB treatment. In fact, N0, P0 and K0 oils showed higher K270 which indicates the presence of oxidized compounds.

**Figure 4:**
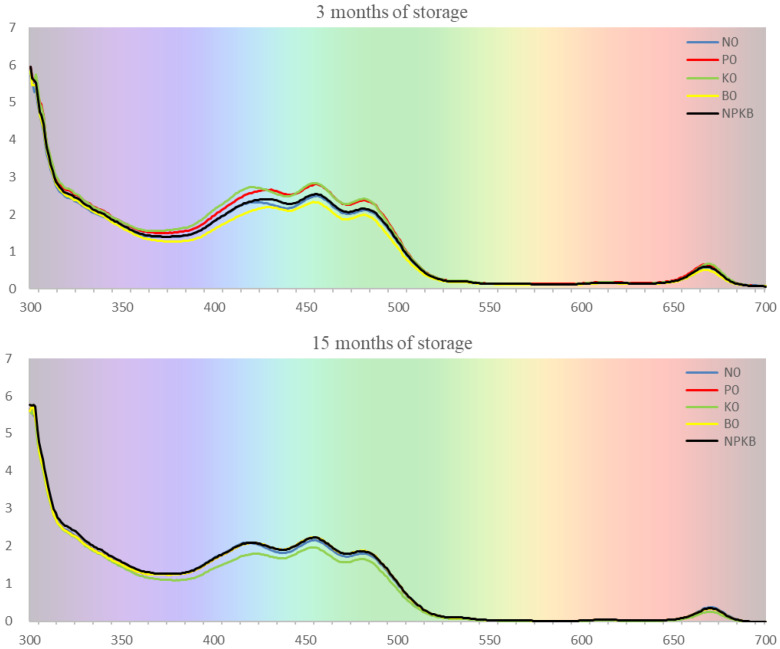
Absorption spectra from olive oil after 3 and 15 months of storage from olive trees supplied with NPKB (control) and without N (N0), P (P0), K (K0) and B (B0) in the harvest of 2018.

The degradation of olive oil between 3 and 15 months of storage in all treatments was also confirmed by the absorption spectra (Figure 4). The spectra of extra-virgin olive oils are characterized by a three-peak band (390–520 nm) and a sharper band (660–675 nm). The first band is due to the overlap between the absorption signals of carotenoids and chlorophylls and the last absorption band is due to the electronic transition of chlorophylls and their derivatives [60]. Pigments such as carotenoids and chlorophyll derivatives are responsible for the color of oil, being a quality index related to oil extraction and olive cultivar [51]. Shendi et al. [61] reported significant changes in the color of several olive oil samples during storage, with fluctuations between redness and yellowness over time. In our study, the oil from the K0 treatment showed greater color degradation from 3 to 15 months of storage. Likewise, it was previously mentioned that this treatment showed higher K270 values (Table 6 and Table 7), indicating greater oxidation. According to Bendini et al. [59] the oxidation of olive oil is an inevitable process that may start after the olives are harvested but certainly during the extraction of the oil, followed by a progressive deterioration that is accentuated during storage.

**Figure 5:**
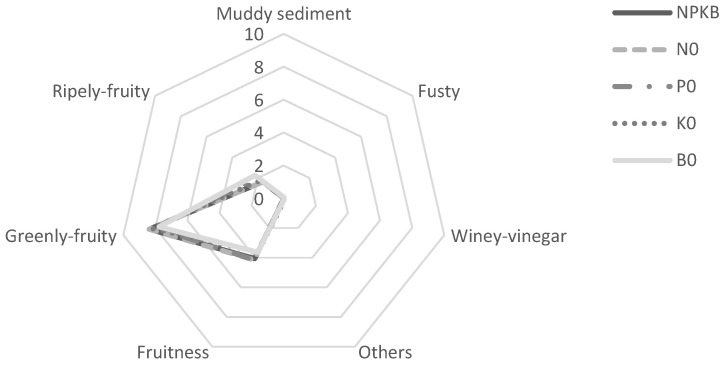
Sensory profiles of virgin olive oils obtained from olive trees supplied with NPKB (control) and without N (N0), P (P0), K (K0) and B (B0) in the harvest of 2018.

The sensory properties of the olive oil were evaluated by a panel of experts using oil samples of 2018 (Figure 5). Its evaluation showed that all the oils tested fell into the medium categories of fruitiness and greenly-fruity. However, some defects were also detected. In olive oil from treatments P0 and B0, the sensorial panel detected muddy sediments and other non-specific defects. In the oil from the K0 treatment, other unidentified defects were detected. The defects detected in a sensorial evaluation are often related to environmental, agronomic and technological factors [46]. Improper management and storage of olives, poor olive oil storage conditions, or oil extracted from damaged or over-ripened fruits can lead to high acidity, low stability and unpleasant sensorial attributes [62,63]. In our study, the muddy sediment detected by the sensory panel may be associated with poor filtration and removal of organic sediments, which would have allowed anaerobic fermentation. This kind of fermentation produces nasty volatile compounds responsible for the muddy sediment defect [59].

## 3. Materials and Methods

### 3.1. Plot Characterization and Experimental Design

The field trial was carried out for three consecutive years (2017–2019) in Bragança (41.807700, −6.733378; 700 m a.s.l.), north-eastern Portugal, in a 7-year-old olive orchard. The olive trees were spaced at 7 m × 6 m and the orchard rainfed managed. The region benefits from a Mediterranean climate, with an average annual air temperature of 12.7 °C and annual rainfall of 772.8 mm. Average monthly temperature, the number of hot and frost days and precipitation and growing degree days during the experimental period are shown in Table 5. The soil is a eutric Regosol, sandy-loam textured (soil separates: 14.5% clay, 27.7% silt and 57.8% sand). Some other soil properties determined from composite soil samples taken at 0–0.20 m just before the trial start are shown in Table 8. The nutritional status of the trees at the beginning of the trial was also evaluated by the elemental composition of leaf samples taken in the winter rest period of 2017. The results are shown in Table 9.

The olive grove plot is very homogeneous in terms of soil fertility and the size of the tree canopy. Thus, the experiment was arranged as a completely randomized design, with five treatments: soil applied N + P + K + B (NPKB, the control treatment); P + K + B (N0); N + K + B (P0); N + P + B (K0); and N + P + K (B0). The experimental design included three replicates, each composed of four similar trees (12 trees per treatment). The control trees received a basal plan of nutrient application with N as ammonium nitrate (34.5% N), P as single superphosphate (18% P_2_O_5_), K as potassium chloride (60% K_2_O) and B as borax (11% B). Fertilizers were applied annually in early spring beneath the trees. N, P, K and B were applied at the rates of 48, 70, 133 and 1.2 g tree^−1^, respectively. In the N0, K0, P0 and B0 plots, the basal plan of nutrient application was also applied with the exception of the nutrient omitted in the respective treatment.

### 3.2. Leaf Water Status and Net Photosynthetic Rate

The physiological measurements determined at the leaf level were performed in the morning on two healthy and well-exposed fully expanded mature leaves per tree in June and September from 2017 to 2019. Leaf water status was estimated from leaves detached and immediately placed into air-tight containers for determining the following variables: (i) fresh weight (FW); (ii) weight at full turgor (TW), measured after the immersion of leaf petioles in demineralized water for 48 h in the dark at 4 °C; and (iii) dry weight (DW), measured after drying at 70 °C to a constant weight. Then, relative water content (RWC) was calculated as RWC (%) = (FW − DW)/(TW − DW) × 100. Net photosynthetic rate (A, μmol CO_2_ m^−2^ s^−1^) was performed using a portable IRGA (LCpro+, ADC, Hoddesdon, UK), operating in the open mode, on cloudless days under natural irradiance and environmental conditions, and assessed using the equation developed by von Caemmerer and Farquhar [64].

### 3.3. Crop Harvest and Oil Extraction

The trees of the experimental design were harvested in November of 2017 and 2019 and in early December of 2018 using a knapsack shaker-machine. The fruits were pulled down, and sheets on the floor were laid to recover them. Thereafter, olive yields were recorded per tree. Samples of fifty olives per experimental unit were randomly collected to determine the biometric variables and maturation index (MI).

For olive oil extraction, 3 kg of healthy fruits per replicate, without any kind of infection or physical damage were used, as described by Brito et al. [47]. Then, the paste was slowly malaxed at about 25 °C for 40 min, centrifuged at 3500× *g* for 10 min, and the oil collected was placed in dark glass bottles and kept at 4 °C for later analysis.

### 3.4. Physical Characterization of Fruits and Maturation Index

Fifty fruits per replicate were randomly selected for the determination of biometric variables, such as pulp and pit fresh weight (FW) and dry weight (DW), and longitudinal and equatorial length. The pulp/pit ratio was estimated as pulp FW/pit FW. The fruit MI was determined according to El Yamani et al. [65] and varied between 0 and 7. Olive fruits were classified into the following categories: 0—olives with intense green epidermis; 1—olives with yellowish-green epidermis; 2—olives with red spots or areas in less than half of the fruit; 3—olives with red or light violet epidermis over more than half of the fruit; 4—olives with black epidermis and totally white pulp; 5—olives with black epidermis and less than half purple pulp; 6—olives with black epidermis and more than half purple pulp; and 7—olives with black epidermis and totally purple pulp. The MI was calculated as follows (a to h—number of fruits in each category):

MI = (a × 0 + b × 1 + c × 2 + d × 3 + e × 4+ f × 5 + g × 6 + h × 7)/*n* (1)

### 3.5. Determination of Fruit Nutritional Variables

Fruit nutritional variables, such as dry matter (DM), organic matter (OM), ash, crude protein (CP), total dietary fiber (TDF) and oil (OC) contents, were determined in lyophilized samples by using the AOAC (Association of Official Agricultural Chemists) methods [66]. The quantification of DM and OM was performed by the methods 934.01 and 946.05, respectively. CP was calculated as Kjeldahl *n* × 6.25 according to the 954.01 method, using a Kjeltec System 1026 Distillation Unit. OC was extracted by the 920.39 method using petroleum ether according to the procedures of the Soxtec System HT equipment. Total dietary fiber was determined using an enzymatic assay procedure (K-TDFR 04/17, Megazyme, Bray, Ireland) following the AOAC procedures (n° 991.3).

Total soluble sugars (TSS) were extracted by heating the samples in 80% ethanol for 1 h at 80 °C according to Irigoyen et al. [67]. Then, the soluble fraction was separated from the solid fraction and the SS concentration determined by the anthrone method. Glucose was used as a standard.

### 3.6. Extraction and Quantification of Polyphenolic Compounds from Olives and Oil

The polyphenolic extraction of olives and oil were adapted from a procedure described by Sousa et al. [68]. Lyophilized olive fruit flesh (300 mg) was dried and mixed with 5 mL MeOH/H_2_O (50:50, *v/v*) and incubated at room temperature for 30 min. After centrifugation at 10,000× *g* for 10 min, the supernatant was removed and reserved in a flask after filtration. This procedure was repeated three times. To remove the fat phase, the mixture was washed three times with 6 mL of hexane and the organic phase was discarded. Extractions were performed in triplicate. Each extract was introduced into a 25 mL round bottom flask, which was filled up to the mark with MeOH/H_2_O (50:50, *v/v*).

For the oil extraction, 2 g of olive oil were mixed with 1 mL of MeOH/H_2_O (70:30, *v/v*). Then, the samples were centrifuged for 10 min at 2800× *g* and the lower phase was carefully removed and reserved in a flask. This procedure was repeated three times and the extractions were performed in triplicate. Each extract was introduced into a 5 mL round bottom flask, which was filled up to the mark with MeOH/H_2_O (70:30, *v/v*).

In the methanolic extract of olives and oil total phenolic compounds (TPC), ortho-diphenol compounds (o-DP), flavonoids (Fl) and total antioxidant capacity (TAC) were determined. The content of TPC was quantified following the Folin–Ciocalteu procedure of Singleton and Rossi [69] and o-DP was determined following the method adapted from Mateos et al. [70]. Both methods used gallic acid as a standard and the results were expressed as mg gallic acid equivalents g^−1^ DW. Fl concentration was determined according to the Barreira et al. [71] modification method, using (+)-catechin as a standard, and the results were expressed as mg (+)-catechin equivalents g^−1^ DW. TAC was determined by the 2,2-azino-bis (3-ethylbenzothiazoline)-6 sulfonic acid (ABTS^+^) radical cation decolorization assay, based on a method described by Barros et al. [72]. A standard curve of the percentage of ABTS^+^ inhibition in the function of Trolox concentration was used for the calculations.

### 3.7. Free Acidity, Peroxide Value and UV Spectrophotometric Indices

Free acidity (%), peroxide index (mg EqO_2_ kg^−1^ oil) and extinctions at specified wavelengths (K232, K270 and ΔK) were determined according to the European Community regulation EEC/2568/91 [54]. The UV-vis absorption spectra of the olive oil samples were performed by using a microplate (SPECTROstar Nano, BMG, Labtech) in the range of 300 and 700 nm at room temperature.

### 3.8. Sensorial Analysis

After six months of storage, a sensorial analysis assay of olive oil was performed by a trained sensory panel following the method for the organoleptic assessment of virgin olive oil [73]. Descriptors were evaluated on a 0–10 intensity scale, with positive attributes such as fruitiness (green or ripe) and negative attributes such as fusty, muddy sediment and winey-vinegary.

### 3.9. Data Analysis

Statistical analysis was performed using the statistical software program SPSS for Windows (v. 20). All data sets satisfied the assumptions of ANOVA based on the homogeneity of variances and normality. The comparison of the effect of the fertilizer treatments in each year was provided by one-way ANOVA. When significant differences were found (α < 0.05), the means were separated by the multiple range Tukey HSD test (α = 0.05).

## 4. Conclusions

The application of N, P, K and B (NPKB treatment) significantly increased olive yield compared to the treatment that did not receive N (N0). The average cumulative olive yield (2017–2019) was the highest in the NPKB treatment, but not significantly different from those obtained in the P0, K0 and B0 treatments. NPKB treatment also influenced positively several biometric variables of the fruits. It showed heavier fruits and higher pulp FW, resulting in a significantly higher pulp/pit ratio. In addition, although depending on the climate conditions of the crop season, the fruits of the NPKB treatment showed a tendency to accumulate more oil.

All olive oil samples were classified in the “extra virgin” category. In all treatments, there was observed a decrease in oil stability from 3 to 15 months of storage, regardless of the treatment, but clearer in N0, P0 and B0 treatments. In the sensorial analysis, all the oils fell into the category medium fruitiness and greenly-fruity. However, some defects were also reported, mainly in treatments P0 and B0, where the sensory panel detected muddy sediment. Thus, this study seems to indicate the importance of N application, but also a balanced nutrient application, and that further studies are needed given the difficulty in finding clear trends in the response of measured variables to fertilizer treatments.

## Figures and Tables

**Table 1:** Leaf concentration of macro and micronutrients from leaf samples taken at the end of the experiment, in December 2019, as a function of fertilizer treatments [with NPKB (control) and without N (N0), P (P0), K (K0) and B (B0)], and respective sufficiency range (SR) for the resting period of olive, after LQARS [22].

	NPKB	N0	P0	K0	B0	*p*-Values	SR
Macronutrients (g k^−1^)							
^1^ Nitrogen	17.7 a	15.7 b	17.5 a	18.4 a	18.0 a	0.0002	16–21
^2^ Phosphorus	1.4 a	1.4 a	1.5 a	1.2 a	1.3 a	0.1828	1–3
^3^ Potassium	7.1 ab	7.8 a	6.5 ab	5.7 b	6.6 ab	0.0350	6–9
^3^ Calcium	4.7 a	4.6 a	4.7 a	4.5 a	4.8 a	0.9613	10–20
^3^ Magnesium	1.2 a	1.3 a	1.3 a	1.4 a	1.3 a	0.2170	1–3
Micronutrients (mg kg^−1^)							
^2^ Boron	29.4 a	25.8 a	27.6 a	27.9 a	18.4 b	<0.0001	15–50
^3^ Iron	78.5 a	94.3 a	80.7 a	86.2 a	92.2 a	0.1172	>40
^3^ Zinc	15.9 a	16.2 a	19.9 a	17.8 a	16.9 a	0.5497	12–35
^3^ Copper	7.4 a	7.0 a	7.7 a	7.7 a	7.4 a	0.8589	5–20
^3^ Manganese	51.1 a	38.8 a	51.4 a	53.6 a	57.3 a	0.1173	20–80

^1^ Kjeldahl; ^2^ Colorimetry; ^3^ Atomic absorption spectrophotometry. Values are means ± SE. In lines, means followed by the same letter are not significantly different by Tukey’s HSD test (α = 0.05).

**Table 2:** Fruit, pulp and pit fresh weight (FW), equatorial length, longitudinal length and pulp/pit ratio (FW) from olive trees supplied with NPKB (control) and without N (N0), P (P0), K (K0) and B (B0) in the harvests of 2017, 2018 and 2019.

		NPKB	N0	P0	K0	B0	*p*-Values
Fruit FW (g)	2017	2.97 ± 0.07	3.01 ± 0.06	3.12 ± 0.09	3.11 ± 0.10	2.97 ± 0.08	0.5266
	2018	3.59 ± 0.09 a	3.15 ± 0.06 bc	3.28 ± 0.07 b	2.93 ± 0.06 c	3.14 ± 0.06 bc	<0.0001
	2019	2.96 ± 0.10	3.16 ± 0.08	2.99 ± 0.11	3.02 ± 0.01	2.95 ± 0.08	0.6585
Equat. Length (mm)	2017	15.2 ± 0.1	15.4 ± 0.1	15.6 ± 0.2	21.6 ± 6.1	15.2 ± 0.2	0.3143
	2018	16.1 ± 0.2 a	15.3 ± 0.1 bc	15.6 ± 0.1 ab	14.9 ± 0.1 c	15.3 ± 0.1 bc	<0.0001
	2019	14.5 ± 0.2	15.2 ± 0.1	14.8 ± 0.2	14.9 ± 0.2	14.8 ± 0.1	0.1355
Long. Length (mm)	2017	21.7 ± 0.2	21.4 ± 0.2	22.1 ± 0.2	21.6 ± 0.3	21.5 ± 0.2	0.1972
	2018	22.9 ± 0.2 a	22.0 ± 0.1 b	22.2 ± 0.2 ab	21.2 ± 0.1 b	22.2 ± 0.2 ab	0.0014
	2019	20.9 ± 0.3	21.7 ± 0.2	21.0 ± 0.3	21.4 ± 0.3	21.1 ± 0.2	0.2705
Pulp FW (g)	2017	2.18 ± 0.06	2.22 ± 0.05	2.35 ± 0.08	2.31 ± 0.09	2.18 ± 0.06	0.3358
	2018	2.84 ± 0.08 a	2.39 ± 0.08 bc	2.48 ± 0.07 b	2.20 ± 0.05 c	2.38 ± 0.06 bc	<0.0001
	2019	2.27 ± 0.08	2.43 ± 0.07	2.25 ± 0.09	2.30 ± 0.09	2.23 ± 0.07	0.5349
Pit FW (g)	2017	0.782 ± 0.019	0.793 ± 0.023	0.768 ± 0.019	0.798 ± 0.021	0.785 ± 0.026	0.9095
	2018	0.756 ± 0.016	0.759 ± 0.015	0.799 ± 0.026	0.730 ± 0.014	0.764 ± 0.014	0.0852
	2019	0.696 ± 0.02	0.735 ± 0.02	0.747 ± 0.03	0.714 ± 0.02	0.729 ± 0.01	0.5777
Pulp/Pit ratio	2017	2.82 ± 0.09	2.85 ± 0.010	3.06 ± 0.08	2.92 ± 0.10	2.82 ± 0.09	0.321
	2018	3.79 ± 0.12 a	3.19 ± 0.11 b	3.18 ± 0.14 b	3.03 ± 0.08 b	3.14 ± 0.11 b	<0.0001
	2019	3.27 ± 0.09	3.32 ± 0.09	3.04 ± 0.11	3.24 ± 0.08	3.06 ± 0.08	0.1183

Values are means ± SE. In lines, means followed by the same letter are not significantly different by Tukey’s HSD test (α = 0.05).

**Table 3:** Physico-chemical composition of olive pulp from olive trees supplied with NPKB (control) and without N (N0), P (P0), K (K0) and B (B0) in the harvests of 2017 and 2018.

		NPKB	N0	P0	K0	B0	*p*-Values
Dry matter (%)	2017	45.2 ± 0.7	44.8 ± 1.4	49.0 ± 1.2	46.7 ± 2.0	49.4 ± 0.5	0.0590
	2018	35.8 ± 1.5 a	34.7 ± 1.7 a	34.8 ± 2.8 a	36.6 ± 0.6 a	26.9 ± 1.3 b	0.0080
Organic matter (%)	2017	92.5 ± 0.6	93.3 ± 0.2	87.3 ± 5.4	93.9 ± 1.0	92.9 ± 0.6	0.3450
	2018	90.8 ± 0.6	89.7 ± 0.9	91.0 ± 0.4	92.5 ± 0.4	89.9 ± 1.6	0.2250
Ash (%)	2017	3.90 ± 0.26	2.84 ± 0.24	2.81 ± 0.39	2.91 ± 0.62	3.19 ± 0.24	0.9170
	2018	1.70 ± 0.18 ab	2.01 ± 0.27 ab	2.33 ± 0.16 b	1.30 ± 0.06 c	3.28 ± 0.27 a	<0.0001
Total dietary fiber (%)	2017	11.9 ± 0.1 ab	12.8 ± 0.3 a	11.5 ± 0.2 b	12.3 ± 0.0.1 ab	12.8 ± 0.2 a	0.0030
	2018	12.2 ± 0.2 ab	12.1 ± 0.3 ab	11.8 ± 0.2 b	13.4 ± 0.4 a	12.6 ± 0.4 ab	0.0130
Crude protein (%)	2017	0.99 ± 0.06 b	2.38 ± 0.36 a	0.94 ± 0.03 b	0.92 ± 0.06 b	2.38 ± 0.04 a	<0.0001
	2018	3.31 ± 0.18 a	2.62 ± 0.07 b	3.19 ± 0.11 ab	3.80 ± 0.19 a	3.26 ± 0.12 a	0.0010
Oil content (%)	2017	55.3 ± 1.1 a	50.1 ± 1.4 a	54.3 ± 1.7 ab	49.9 ± 2.2 b	50.9 ± 1.6 ab	0.0410
	2018	46.7 ± 2.0 ab	49.0 ± 2.0 ab	50.0 ± 1.3 a	43.4 ± 1.2 b	50.7 ± 1.1 a	0.0110
Soluble sugars (mg g^−1^)	2017	98.2 ± 2.0 b	108.9 ± 1.9 ab	109.4 ± 1.7 a	111.0 ± 4.4 a	101.3 ± 1.4 ab	0.0090
	2018	157.2 ± 6.0 a	141.2 ± 1.5 b	135.3 ± 2.4 b	158.9 ± 5.1 a	134.4 ± 1.5 b	<0.0001

Values are means ± SE. In lines, means followed by the same letter are not significantly different by Tukey’s HSD test (α = 0.05).

**Table 4:** Total phenols (TP), ortho-diphenols (o-DP), flavonoids (Fl) and total antioxidant capacity (TAC) on olives and olive oil from olive trees supplied with NPKB (control) and without N (N0), P (P0), K (K0) and B (B0) in the harvests of 2017 and 2018.

	Olive Fruit				Olive Oil			
	TP (mg g^−1^ DW)	o-DP (mg g^−1^ DW)	Fl (mg g^−1^ DW)	TAC (mmol g^−1^ DW)	TP (mg kg^−1^ DW)	o-DP (mg kg^−1^ DW)	Fl (mg kg^−1^ DW)	TAC (mmol kg^−1^ DW)
2017								
NPKB	26.6 ± 0.7 d	24.3 ± 0.9 b	24.9 ± 1.3 c	91.8 ± 2.0 b	116.2 ± 1.0 cd	54.6 ± 0.5 d	61.9 ± 2.4 c	66.1 ± 1.5 c
N0	31.3 ± 0.9 c	29.0 ± 0.8 ab	27.2 ± 2.9 bc	102.0 ± 3.0 b	132.3 ± 1.4 b	71.8 ± 0.8 a	73.3 ± 2.1 bc	73.9 ± 2.4 bc
P0	35.5 ± 0.7 ab	32.7 ± 1.3 a	38.3 ± 4.6 a	120.1 ± 2.1 a	125.1 ± 1.6 bc	59.5 ± 0.8 c	78.9 ± 2.8 b	81.4 ± 2.4 b
K0	38.1 ± 1.5 a	31.5 ± 2.4 a	38.0 ± 3.6 a	114.6 ± 3.9 a	143.6 ± 3.7 a	67.7 ± 1.5 b	105.6 ± 4.6 a	96.5 ± 3.1 a
B0	34.2 ± 0.9 bc	28.1 ± 1.4 ab	35.7 ± 4.4 ab	102.4 ± 2.2 b	115.4 ± 2.5 d	57.1 ± 0.7 cd	75.6 ± 3.2 b	74.8 ± 1.3 bc
*p*-values	<0.0001	0.0030	0.0200	<0.0001	<0.0001	<0.0001	<0.0001	<0.0001
2018								
NPKB	29.0 ± 1.0 b	37.9 ± 2.2 ab	25.7 ± 3.0	67.9 ± 4.0 b	170.5 ± 1.2 c	88.5 ± 1.4 b	109.2 ± 2.0 c	122.4 ± 3.1 c
N0	34.4 ± 0.3 a	43.7 ± 2.5 a	29.9 ± 4.3	90.5 ± 2.1 a	229.4 ± 4.9 a	102.8 ± 1.1 a	143.5 ± 3.1 a	179.5 ± 3.3 a
P0	28.1 ± 0.4 b	45.4 ± 2.0 a	23.4 ± 1.6	94.2 ± 3.2 a	183.0 ± 7.2 bc	84.9 ± 3.5 b	102.1 ± 2.9 c	125.2 ± 3.6 c
K0	24.9 ± 0.5 c	32.9 ± 2.3 b	20.5 ± 1.4	73.3 ± 1.7 b	199.6 ± 4.4 b	99.6 ± 1.3 a	121.1 ± 2.4 b	160.0 ± 4.3 b
B0	26.8 ± 0.5 bc	43.6 ± 1.7 a	25.3 ± 4.5	89.0 ± 1.5 a	190.2 ± 2.5 b	89.7 ± 1.1 b	106.7 ± 1.7 c	125.6 ± 1.8 c
*p*-values	<0.0001	0.0010	0.3620	<0.0001	<0.0001	<0.0001	<0.0001	<0.0001

Values are means ± SE. In columns, means followed by the same letter are not significantly different by Tukey’s HSD test (α = 0.05).

**Table 5:** Average monthly temperature (maximum, ATX, minimum, ATN, and mean, ATE), maximum (TX) and minimum (TN) air temperature, number of hot days (HD, maximum temperature above 35 °C) and frost days (FD, minimum temperature below 0 °C), precipitation and growing degree days (GDD, assuming that the air temperature threshold below that which the plants’ growth does not progress is 7 °C [49] during the experimental period (2017–2019).

	ATX (°C)	ATN (°C)	ATE (°C)	TX (°C)	HD	TN (°C)	FD	Rainfall (mm)	Accumulated Rainfall (mm)	GDD (°C)	Accumulated GDD (°C)
2017											
January	9.6	−0.8	4.4	17.7		−7.7	18	51.8	51.8	10.1	10.1
February	12.5	3.3	7.9	19.6		−2.0	2	163.8	215.6	39.3	49.3
March	15.4	4.2	9.8	24.0		−1.1	3	47.2	262.9	107.3	156.6
April	21.0	5.7	13.4	26.6		−0.7	1	13.0	275.8	191.8	348.4
May	22.7	10.4	16.5	31.2		3.5		51.8	327.6	295.7	644.1
June	29.0	14.3	21.7	38.1	4	6.9		4.6	332.2	439.6	1083.7
July	30.7	14.0	22.3	36.2	4	9.7		7.1	339.3	474.6	1558.3
August	30.1	14.1	22.1	36.6	1	7.1		5.1	344.4	468.7	2027.0
September	25.8	10.0	17.9	31.6		2.6		0.0	344.4	326.5	2353.5
October	24.2	8.0	16.1	31.6		2.0		14.0	358.4	280.9	2634.3
November ^a^	16.1	2.2	9.1	18.5		−2.7	4	24.1	382.5	38.6	2672.9
2017 ^a^	21.6	7.8	14.6	38.1	9	−7.7	28				
November	14.7	1.5	8.0	18.5		−4.9	11	37.3	419.8	51.4	2724.2
December	10.3	0.7	5.5	14.5		−5.7	16	110.0	529.8	21.0	2745.2
2017			13.5	38.1	9	−7.7	51				
2018											
January	10.2	1.7	6.0	15.5		−4	9	50.8	50.8	21.9	21.9
February	10.4	−0.7	4.8	16.2		−7.2	18	92.7	143.5	36.5	58.4
March	10.1	2.5	6.3	19.5		−3.7	3	193.8	337.3	14.0	72.4
April	16.2	5.8	11.0	27.1		0.2		271.0	608.3	143.3	215.7
May	20.9	8.4	14.7	27.7		1.8		38.6	646.9	238.2	453.8
June	24.5	12.8	18.7	32.5		6.5		124.2	771.1	353.5	807.3
July	27.3	14.2	20.8	31.7		11.2		26.16	797.2	427.1	1234.4
August	31.7	15.0	23.3	39.2	5	10.7		0	797.2	387.0	1621.4
September	28.9	14.3	21.6	31.6		10.4		2.0	799.3	387.0	2008.3
October	19.3	7.5	13.4	28.6		2.1		49.3	848.5	200.9	2209.2
November	9.4	3.2	6.3	19.0		−1.2	3	137.9	986.4	29.1	2238.2
December ^a^	10.7	5.4	8.1	12.2		2.8		0	986.4	3.1	2241.3
2018 ^a^	18.3	7.5	12.9	39.2	5	−7.2	33				
December	10.8	3.3	7.1	16.5		−1.2	5	52.3	1038.8	29.8	2271.1
2018			12.5	39.2	5	−7.2	38				
2019											
January	10.4	−0.4	5.0	15.2		−4.9	20	32.5	32.5	7.8	7.8
February	13.6	1.1	7.3	20.5		−4	8	72.4	104.9	32.5	40.2
March	16.7	3.1	9.9	21.1		−1.7	2	21.1	126.0	91.6	131.8
April	15.4	5.0	10.2	25		−0.2	1	114.8	240.8	110.1	241.9
May	23.7	8.3	16.0	31.2		5.1		9.2	249.9	241.7	483.6
June	23.8	9.8	16.8	33.5		2.2		39.6	289.6	296.0	779.5
July	30.1	14.8	22.5	36.5	1	9.4		22.11	311.7	479.5	1259.0
August	28.9	14.2	21.6	33.2		6.7		23.37	335.0	451.4	1710.4
September	25.5	11.7	18.6	32		5.3		41.9	377.0	344.6	2054.9
October	19.4	8.7	14.1	28.7		1.1		47.8	424.7	219.0	2273.9
November ^a^	11.5	5.8	8.7	18.5		1.8		52.32	477.0	36.2	2310.0
2019 ^a^	19.9	7.5	13.7	36.5		−4.9	31				
November	11.6	5.6	8.6	18.5		−1.9	1	172.0	649.0	97.4	2407.4
December	10.5	2.7	6.6	15.5		−3.4	10	235.5	884.4	25.3	2432.7
2019			12.8	36.5		−4.9	42				

^a^ until harvest.

**Table 6:** Quality indices of olive oil from olive trees supplied with NPKB (control) and without N (N0), P (P0), K (K0) and B (B0) evaluated 3 months after the harvests of 2017 and 2018.

		NPKB	N0	P0	K0	B0	*p*-Values
Free acidity (%)	2017	0.046 ± 0.023	0.117 ± 0.022	0.136 ± 0.031	0.116 ± 0.022	0.048 ± 0.024	0.0750
	2018	0.140 ± 0.012	0.158 ± 0.012	0.127 ± 0.007	0.160 ± 0.001	0.151 ± 0.006	0.0950
Peroxide index (mEq O_2_ kg^−1^)	2017	8.09 ± 0.30 a	5.42 ± 0.09 b	6.80 ± 1.40 ab	5.03 ± 0.22 b	5.62 ± 0.10 b	0.0450
	2018	7.99 ± 0.81	10.30 ± 0.20	10.90 ± 0.30	9.19 ± 1.58	9.24 ± 1.07	0.3000
K232	2017	1.12 ± 0.14	0.94 ± 0.05	1.14 ± 0.126	0.99 ± 0.08	1.09 ± 0.202	0.7690
	2018	2.18 ± 0.14	2.39 ± 0.20	2.38 ± 0.20	2.37 ± 0.05	2.28 ± 0.07	0.8140
K270	2017	0.079 ± 0.010 b	0.066 ± 0.004 b	0.085 ± 0.007 ab	0.116 ± 0.002 a	0.079 ± 0.010 b	0.0080
	2018	0.186 ± 0.006 a	0.164 ± 0.0003 b	0.171 ± 0.0003 ab	0.185 ± 0.001 a	0.183 ± 0.0003 a	0.0090
ΔK	2017	0.004 ± 0.001 ab	0.003 ± 0.000 b	0.004 ± 0.001 ab	0.006 ± 0.001 a	0.003 ± 0.001 b	0.0040
	2018	0.031 ± 0.023	0.006 ± 0.000	0.006 ± 0.0003	0.006 ± 0.0007	0.006 ± 0.0003	0.3860

Values are means ± SE. In lines, means followed by the same letter are not significantly different by Tukey’s HSD test (α = 0.05).

**Table 7:** Quality indices of olive oil (samples from the harvest of 2018 after 15 months of storage) from olive trees supplied with NPKB (control) and without N (N0), P (P0), K (K0) and B (B0).

	Month	NPKB	N0	P0	K0	B0	*p*-Values
Peroxide index (meq O_2_ kg^−1^)	3	7.99 ± 0.81	10.27 ± 0.2	10.91 ± 0.31	9.19 ± 1.58	9.24 ± 1.07	0.3001
	15	5.94 ± 0.55	7.50 ± 0.25	7.28 ± 0.86	7.41 ± 0.38	7.09 ± 0.66	0.3679
K232	3	2.18 ± 0.14 b	2.39 ± 0.20 b	2.38 ± 0.19 a	2.37 ± 0.05 b	2.28 ± 0.07 b	0.8140
	15	4.29 ± 0.19 b	4.41 ± 0.24 b	4.82 ± 0.64 a	5.54 ± 0.55 b	5.26 ± 0.56 c	<0.0001
K270	3	0.186 ± 0.009 a	0.165 ± 0.003 b	0.171 ± 0.004 b	0.184 ± 0.0009 a	0.183 ± 0.003 a	<0.0001
	15	0.266 ± 0.001 b	0.299 ± 0.006 b	0.312 ± 0.004 a	0.302 ± 0.012 b	0.289 ± 0.001 c	<0.0001
ΔK	3	0.032 ± 0.023 b	0.005 ± 0.00005 b	0.006 ± 0.0002 a	0.006 ± 0.0005 b	0.006 ± 0.0002 b	<0.0001
	15	0.0019 ± 0.0001 b	0.0032 ± 0.00002 b	0.0005 ± 0.00003 a	0.0017 ± 0.0001 b	0.0015 ± 0.0001 b	<0.0001

Values are means ± SE. In lines, means followed by the same letter are not significantly different by Tukey’s HSD test (α = 0.05).

**Table 8:** Selected soil properties from composite samples (10 cores per composite sample, n = 3) taken from the 0–0.20 m soil layer at the beginning of the experiment.

Soil Properties	
^1^ Organic carbon (g kg^−1)^	5.7 ± 0.10
^2^ pH_(H2O)_ (1:2.5)	8.9 ± 0.46
^3^ Extractable P (mg kg^−1^, P_2_O_5_)	63.2 ± 7.02
^3^ Extractable K (mg kg^−1^, K_2_O)	90.0 ± 7.41
^4^ Exchangeable Ca (cmol_c_ kg^−1^)	6.7 ± 0.68
^4^ Exchangeable Mg (cmol_c_ kg^−1^)	2.2 ± 0.16
^4^ Exchangeable K (cmol_c_ kg^−1^)	0.2 ± 0.02
^4^ Exchangeable Na (cmol_c_ kg^−1^)	0.4 ± 0.04
^4^ Exchangeable acidity (cmol_c_ kg^−1^)	0.15
Cation exchange capacity (cmol_c_ kg^−1^)	10.5 ± 0.85

^1^ Wet digestion (Walkley-Black); ^2^ Potenciometry (soil:solution, 1:2.5); ^3^ Ammonium lactate (Egner-Riehm); ^4^ Ammonium acetate, (pH 7.0).

**Table 9:** Leaf concentration of macro and micronutrients (mean ± standard deviation, *n* = 3) from leaf samples taken in January 2017, and the respective sufficiency ranges (SR) for the resting period of olive, after LQARS [22].

Macronutrients (g kg^−1^)		SR	Micronutrients (mg kg^−1^)		SR
^1^ Nitrogen	19.4 ± 0.88	16–21	^2^ Boron	34.8 ± 2.38	15–50
^2^ Phosphorus	1.3 ± 0.23	1–3	^3^ Iron	86.1 ± 10.98	>40
^3^ Potassium	6.4 ± 1.11	6–9	^3^ Zinc	11.1 ± 1.51	12–35
^3^ Calcium	5.2 ± 0.62	10–20	^3^ Copper	5.9 ± 0.91	5–20
^3^ Magnesium	1.1 ± 0.20	1–3	^3^ Manganese	47.3 ± 8.02	20–80

^1^ Kjeldahl; ^2^ Colorimetry; ^3^ Atomic absorption spectrophotometry.

## Data Availability

The data presented in this study are available on request from the corresponding author.
